# Prevalence and Molecular Characterization of Ascaridoid Parasites of Philippine *Decapterus* Species

**DOI:** 10.2478/jofnem-2022-0030

**Published:** 2022-08-14

**Authors:** Tres Tinna M. Dela Cruz, Kennesa Klariz R. Llanes, Joliesa Mae S. Toledo, Junard A. Catabay, Raffy Jay C. Fornillos, Ian Kendrich C. Fontanilla, Vachel Gay V. Paller

**Affiliations:** 1 Institute of Biological Sciences, University of the Philippines Los Baños, Pedro R. Sandoval Avenue, Los Baños, Laguna 4031, Philippines; 2 Science Department, College of Natural Sciences and Mathematics, Mindanao State University-General Santos, General Santos City, South Cotabato 9500, Philippines; 3 Biology Department, College of Arts and Sciences, Central Mindanao University, Musuan, Maramag, Bukidnon 8714, Philippines; 4 Institute of Fisheries and Aquatic Sciences, Bataan Peninsula State University-Orani Campus, Bataan 2112, Philippines; 5 Institute of Biology, College of Science, University of the Philippines, Diliman, Quezon City, 1101, Philippines

**Keywords:** *Anisakis typica*, *Decapterus*, host–parasite relationship, molecular characterization, *Raphidascaris* (*Ichthyascaris*) *lophii*

## Abstract

There are relatively few studies on parasite fauna of marine fishes in Philippine waters. This study aimed to determine the prevalence of marine ascaridoid infection in *Decapterus* species in Balayan Bay and Tayabas Bay. A total of 371 fishes belonging to three different species of *Decapterus* (*D. tabl* [*n* = 130], *D. macrosoma* [*n* = 121], and *D. maruadsi* [*n* = 120]) were collected. Ascaridoid parasite larvae were found in all fish host species, with an overall fish infection rate of 22%. The highest infection rate was observed in *D. tabl* (27.69%), followed by *D. macrosoma* (19%), and then *D. maruadsi* (17.50%). Moreover, a higher prevalence of infection was detected in Tayabas Bay (27.57%) than in Balayan Bay (15.59%). Molecular analyses based on the *ITS2* and *18S rRNA* gene supported the identification of the larvae into two species: *Anisakis typica* and *Raphidascaris* (*Ichthyascaris*) *lophii*. This is the first report of the genetic identification of these two helminth parasites in *Decapterus* fish species in the Philippines. Paucity in the database of Philippine marine fish parasites warrants more research efforts, especially concerning economically important fish species with implications to food safety and food security.

## Highlights

- Three hundred seventy-one *Decapterus* spp. (scad fish) were collected from Balayan Bay and Tayabas Bay of the southern Luzon, Philippines.
- Twenty-two percent of the fish samples were parasitized with Anisakidae and Raphidascarididae.
- Phylogenetic analysis using ITS2 and 18S gene markers demonstrated the species identification of *Anisakis typica* and *Raphidascaris* (*Ichthyascaris*) *lophii*.

Fishes are important food sources that provide nutrition as well as livelihood to millions of people worldwide. The Food and Agriculture Organization (FAO) of the United Nations has documented that 88% of the global fish production in 2016 was intended for human consumption, surpassing that of the meat from terrestrial animals (FAO, 2018). Moreover, the fisheries sector is recognized as a large part of the trade industry for food commodities, with high export rates from developing countries such as the Philippines (FAO, 2020).

Filipinos are generally fish eaters because of the rich aquatic resources throughout the archipelago. One of the staple sources of proteins are the round scad fish of the family Carangidae (Rafinesque, 1815), genus *Decapterus* (Bleeker, 1851). They are locally known as “*galunggong*” which are traditionally associated with the diet of Filipinos belonging to the low- and middle-class income groups. The round scad fishery contributed to 202.66 thousand metric tons (MT) in volume of commercial fisheries production in the country, which is equivalent to 4.6% of the total fishery production in 2020 (PSA, 2021). Being widely available in Philippine waters, these fish species are usually subjected to heavy exploitation by commercial fishing (DA-BFAR, 2018). Heavy fishing and other anthropogenic factors greatly influence fish stocks of both farm and wild populations (Moiseenko et al., 2010; Azimi and Rocher, 2016). On the one hand, parasites and parasitic diseases have tremendous effects on the fish host populations; however, evidence for such interactions is rare (Azimi and Rocher, 2016). Studies on marine parasites focused on the use of parasites as natural tags for stock assessment (Mattiucci et al., 2008) and as biological indicators of pollution (Adams et al., 1989; Williams et al., 1992; Dzikowski et al., 2003; Williams and Mackenzie, 2003; Sures, 2004; Malek et al., 2007).

According to the studies from American, European, and Mediterranean countries, more than 100,000 species of fish parasites have been estimated to be present globally (Mishra and Chubb, 2009; Gibson et al., 2014; Costa et al., 2017). Parasites of marine pelagic fishes are very diverse and serve a very important role in the ecological perspective (Poulin and Morand, 2004). More importantly, fish parasitological studies shed light on zoonotic parasites that cause potent problems to human health. In the Philippines, studies on marine parasitology are scarce. The only published checklist of Philippine fish parasites by far was made available by Arthur and Lumanlan-Mayo (1997). Velasquez (1972) reported infection in marine fishes sold in Manila and nearby provinces. Furthermore, a survey on the parasites of several fish species from the Visayas region in the early 1990s was conducted. Parasites identified were microsporidia, cestodes, and nematodes, and all of which are found in low prevalence, posing low risks of infection. Reports of *Anisakis typica*, *A. brevispiculata*, and *A. paggiae* in a whale host were recorded in the Philippines (Quaizon, 2016). A majority of the definitive hosts of ascaridoid parasites are cetaceans. Cetaceans such as dolphins and whales frequently migrate to Philippine waters to feed. Marine fishes such as *Decapterus* spp., *Sardinella* spp., and other pelagic fishes are the intermediate hosts of ascaridoid parasites, in which some of the commercially important fishes can be infected. Currently, there is no update on the parasitic helminth of *Decapterus* spp., which is a commercially important fish food in the Philippine (Petersen et al., 1993).

This study aimed to determine the prevalence of ascaridoid nematode infection in *Decapterus* spp. collected from Tayabas Bay and Balayan Bay in the southern part of Luzon, Philippines. The presence of ascaridoid anisakid parasites was confirmed using molecular analysis. Indices such as prevalence, mean intensity, and parasite aggregation in hosts were considered.

## Materials and Methods

### Ethical considerations

Ethical approval for animal use was obtained from the Institutional Animal Care and Use Committee (IACUC) University of the Philippines, Los Baños (Protocol No: CAS-2019-001). Approval and sampling were coordinated to the National Stock Assessment Program (NSAP) of the Bureau of Fisheries and Aquatic Resources Region IV-A (BFAR-4A) in Lucena City Regional Office.

### Collection of fish samples

Balayan Bay (13.8447° N, 120.8499° E) and Tayabas Bay (13.5999° N, 121.7195° E) are among the productive fishing grounds of the Philippines. These bays are adjacent to the West Philippine Sea and are part of the Verde Island Passage, which is considered to be rich in marine biodiversity. These two bays are included in Fisheries Management Area 12 (FMA 12) designated by the Bureau of Fisheries and Aquatic Resources (BFAR) for conservation and management of fisheries in the Philippines.

**Figure 1:**
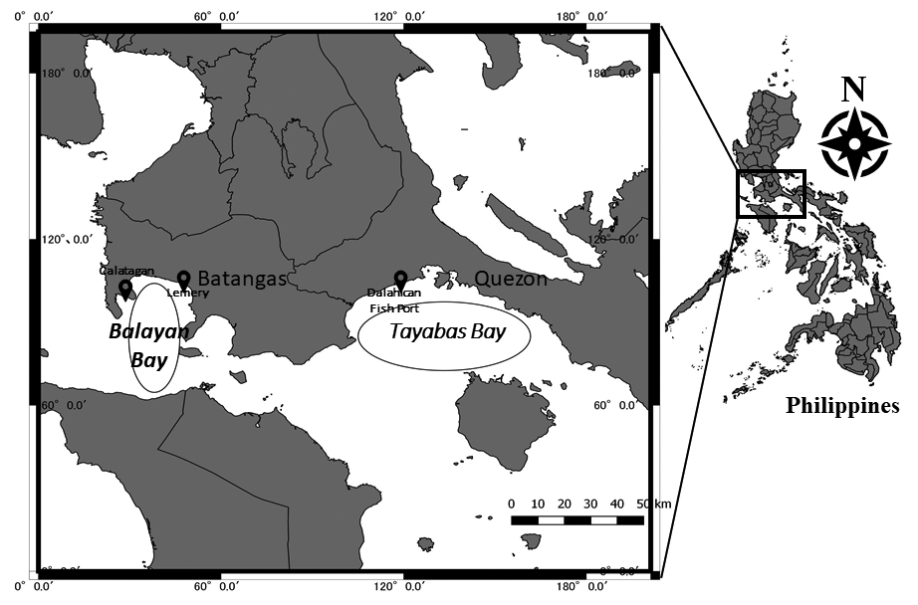
Fish landing area at Balayan Bay and Tayabas Bays, southern Luzon, Philippines, for fish sample collection.

Fish samples were obtained from two fish landing areas in Batangas and Lucena in May 2018. The Batangas fish landing area located in Lemery and Calatagan has most of their fish catch from the adjacent Balayan Bay. The fish landing in Dalahican Port in Lucena acquires most of the fishes and other marine resources from Tayabas Bay (Fig. 1).

A total of 371 fish samples were collected from Tayabas Bay and Balayan Bay and were identified based on key morphological characteristics found in FishBase (Froese and Pauly, 2019). Three species of *Decapterus* were identified in the two sampling sites, namely, *D. maruadsi* (*n* = 120), *D. tabl* (*n* = 130), and *D. macrosoma* (*n* = 121).

The fish samples were maintained in cold temperature and immediately transported to the Parasitology Research Laboratory, Institute of Biological Sciences, University of the Philippines Los Baños (UPLB) for dissection and processing. Before dissection, the total body length and weight of individual fish samples were measured. Sex of fish samples was also recorded based on observation of the gonads following dissection (Ohshimo et al., 2014).

### Parasite collection and processing

The body cavity, stomach, and intestine of each dissected fish were carefully examined with the naked eye and under a stereomicroscope (Leica EZ4; Leica Microsystems Pte Ltd, Singapore) for the presence of helminth larvae (third-stage larvae of nematodes). The helminths were removed, counted, and preserved in 70% ethanol in separate Eppendorf tubes. These larvae were then cleared in a mixture of 5% glycerin in 70% ethanol for further examination under a compound microscope (Nikon, Tokyo, Japan). Parasites were identified based on morphological characteristics (Mattiucci et al., 2005; Borges et al., 2012; Hien et al., 2021). The specimens were photographed using digital microscope cameras OptixCam Summit Series SK2-5.2Xx USB (Microscope LLC, VA, USA) and Optika version 2.1 (Optika Microscopes, Bergamo, Italy). All parasites were removed and preserved in 70% ethanol, but only representative individuals of those initially identified as marine ascaridoid parasites through light microscopy were subjected to molecular processing for species identification.

### DNA extraction and PCR amplification

Fifteen (five per fish species) morphologically similar ascaridoid larvae were randomly selected for molecular identification. Genomic DNA was extracted from ethanol-fixed midsections of individual larvae using a DNeasy^TM^ Tissue Kit (Qiagen, Hilden, Germany). Identification of the parasites was carried out through analysis of the small subunit ribosomal ribonucleic acid sequences. Primers were designed using the Primer-BLAST (Primer3web v. 4.1.0) tool (Ye et al., 2012) to target the 18S rRNA gene and the internal transcribed spacer 2 (ITS2) region, flanked by partial sequences of 5.8S and 28S genes. The 18S gene marker was amplified using the primer pair SSU-F (5'-ATGAGAGGGCAAGTCTGGTG-3') and SSU-R (5'-CTGTCAATCCTCACGGTGTC-3'). The amplification reactions for this gene were performed in a 25 ml volume mastermix containing 2.5 ml 10X PCR buffer, 1.875 ml 10 mM dNTPs, 2 ml 25mM MgCl_2_, 1 ml 10 mM each of forward and reverse primers, 0.125 U Taq polymerase, and 2 ml 40ng/ml DNA template. The PCR conditions included an initial denaturation at 94°C for 4 min, followed by 40 cycles of denaturation at 94°C for 1 min, annealing at 55°C for 30 s, extension at 65°C for 1 min, and a final extension step at 72°C for 10 min. The 5.8S-ITS2-28S region was amplified using forward primer AscF (5'-GATCGATGAAGAACGCAGCC-3') and reverse primer AscR (5'-TTTGCAACTTTCCCTCACGG-3') with the following PCR conditions: initial denaturation of 94°C for 4 min, followed by 40 cycles of denaturation at 94°C for 1 min, annealing at 60°C for 30 s, and extension at 65°C for 1 min. A final extension step was added at 72°C for 10 min. The PCR was performed in a 25 ml volume with a similar composition of the PCR mastermix for the 18S gene amplification. The PCR products were resolved using a 1% agarose gel stained with 1% EtBr run in a horizontal gel electrophoresis system at 100 V for 30 min. Bands were visualized using a UV transilluminator. Distinct bands were sliced out from the gel and were stored in a properly labeled 2-ml microcentrifuge tube ready for gel extraction.

### Purification and DNA sequence analysis

From the PCR amplification, 14 amplicons with a molecular weight of about 1 kb were purified using a QIAquick Gel Extraction Kit (Qiagen, Germany) and were sent to Macrogen, South Korea, for standard Sanger sequencing. Sequence assembly was performed using the Staden Package 4.0 (Staden et al., 2000). The generated consensus sequences were subjected to local alignment using the Basic Local Alignment Search Tool (https://blast.ncbi.nlm.nih.gov/Blast.cgi?PAGE_TYPE=BlastSearch) to identify the closest species matches (Altschul et al., 1990). Sequences from families Anisakidae and Raphidascaridae with closest species matches from BLAST were obtained from GenBank (Table 1). The aligned sequences of 18S and ITS2 were edited and trimmed in BioEdit Sequence Alignment Editor (Hall, 1999). The final sequence alignment for each marker was converted to a PAUP/NEXUS file in Data Analysis in Molecular Biology and Evolution (DAMBE) (Xia, 2013). The optimal model of evolution was chosen according to the Akaike Information Criterion (AIC) using jModelTest 1 (Darriba et al., 2012). In the 18S final sequence dataset, the TrNeF + G model (Tamura and Nei, 1993) was implemented involving 34 nucleotide sequences, with a total of 628 positions. And in the ITS2 final sequence dataset, the HKY + G model (Hasegawa et al., 1985) was implemented involving 33 nucleotide sequences, with a total of 460 positions. The phylogenetic relationships between nematodes were reconstructed using neighbor-joining (NJ) trees (Saitou and Nei, 1987) in PAUP version 4.0b10 (Swofford, 2002) and maximum-likelihood (ML) (Felsenstein, 1981) trees in PhyML version 3.0 (Guindon and Gascuel, 2003). The outgroup used in the reconstructed 18S phylogenetic tree was *Ascaris lumbricoides* (Linnaeus, 1758) from the family Ascarididae (Baird, 1853). For ITS2, *Raphidascaris (Sprentascaris) andersoni* (Malta et al., 2018) from the family Raphidascarididae (Hartwich, 1954) was used as an outgroup. Bootstrap resampling for both NJ and ML trees computed for 1,000 replicates and all bootstrap values >50 are shown.

**Table 1:** GenBank sequences of close species matches used for phylogeny construction.

Ascaridoid species	Host	Habitat	Geographical Origin	ITS2	18S rRNA
*Anisakis pegreffii*	*Trachinus radiatus*	Marine	Tunisia	MT820022	-
*Anisakis pegreffii*	*Conger myriaster*	Marine	China	-	MF072697
*Anisakis pegreffii*	*Caretta*	Marine	Italy	-	EF180082
*Anisakis simplex*	*Globicephala melaena*	Marine	Norway	AY826723	-
*Anisakis simplex*	*Scomber japonicus*	Marine	Japan	LC621351	-
*Anisakis simplex*	*Carcharhinus sorrah*	Marine	China	-	MF072711
*Anisakis typica*	*Tursiops aduncus*	Marine	Egypt	HF911524	-
*Anisakis typica*	Bottlenose dolphin	Marine	China	KF673776	-
*Anisakis typica*	*Rastrelliger kanagurta*	Marine	Thailand	AB432909	-
*Anisakis typica*	*Stenella longirostris*	Marine	Brazil	AY826724	-
*Anisakis typica*	*Katsuwonus pelamis*	Marine	Indonesia	KC928262	-
*Anisakis typica*	*Sotalia fluviatilis*	Marine	Brazil	EU327686	-
*Anisakis ziphidarum*	*Pagellus bogaraveo*	Marine	Portugal	JN005767	-
*Anisakis ziphidarum*	*Hoplostethus cadenati*	Marine	Mauritius	EU718473	-
*Anisakis ziphidarum*	*Ziphius cavirostris*	Marine	South Africa	AY826725	-
*Contracaecum eudyptulae*	*Eudyptula minor*	Marine	Australia	-	EF180072
*Contracaecum microcephalum*	NM	NM	NM	-	AY702702
*Contracaecum multipapillatum*	NM	NM	NM	-	U94370
*Hysterothylacium aduncum*	*Zoarces viviparus*	Marine	Denmark	JX845137	-
*Hysterothylacium aduncum*	Dolphin fish	Marine	South Korea	HQ702733	-
*Hysterothylacium aduncum*	*Lophius litulon*	Marine	China	-	MF072693
*Hysterothylacium deardorffoverstreetorum*	*Paralichthys isosceles*	Marine	Brazil	-	JF718550
*Hysterothylacium fortalezae*	NM	NM	NM	-	U94374
*Hystetothylacium reliquens*	NM	NM	NM	-	U94376
*Hystetothylacium tetrapteri*	*Kajikia audax*	Marine	China	-	MF072705
*Hysterothylacium thalassini*	*Priacanthus macracanthus*	Marine	China	-	MF072702
*Pseudanisakis rajae*	*Raja pulchra*	Marine	China	-	MF072707
*Pseudoterranova decipiens*	*Gadus morhua*	Marine	Denmark	KM273088	-
*Pseudoterranova decipiens*	NM	NM	NM	-	U94380
*Raphidascaroides brasiliensis*	*Platydoras costatus*	Freshwater	Brazil	-	KP726276
*Raphidascaroides moraveci*	*Platydoras armatulus*	Freshwater	Brazil	-	KP726278
*Raphidascaris acus*	*Esox lucius*	Freshwater; brackish water	Finland	-	DQ503460
*Raphidascaris lanfredae*	*Geophagus maximus*	Freshwater	Brazil	-	KX859077
*Raphidascaris longispicula*	NM	Marine	South China Sea	JN102362	-
*Raphidascaris longispicula*	*Uroconger lepturus*	Marine	China	KP326546	MF072704
*Raphidascaris lophii*	NM	NM	China	MF422212	-
*Raphidascaris lophii*	*Lophius litulon*	Marine	China	MH211584	MF072692
*Raphidascaris lophii*	NM	Marine	China	JF809816	-
*Terranova caballeroi*	NM	NM	NM	-	U94381
^a^ *Raphidascaris (Sprentascaris) andersoni*	*Gymnogeophagus balzanii*	Freshwater	Brazil	MK141032	-
^a^ *Ascaris lumbricoides*	NM	NM	NM	**-**	U94366

a Outgroup species ITS2, internal transcribed spacer 2; NM, not mentioned.

### Data analysis

The prevalence of parasite infection was calculated by dividing the number of infected fish by the total number of fish examined, multiplied by 100, and expressed as percentage. Mean intensity was calculated by dividing the total number of parasites by the total number of infected fish hosts examined. On the other hand, chi-square tests were performed to determine variations in helminth prevalence and mean intensities among fish host species and between sampling sites. This was performed using IBM SPSS Statistics v. 25 with 95% confidence interval and 0.05 level of significance. Parasite aggregation in the fish host population was measured using Poulin’s discrepancy index (*D*), considering the *v* variance/mean ratio (s^2^/m), and *k* value of negative binomial distribution, with values ranging from 0 (no aggregation) to 1 (theoretical maximum) (Estaño et al., 2021). Aggregation analysis to generate frequency distribution of the helminths was done in Quantitative Parasitology (QPweb) (Reiczigel et al., 2019).

## Results

### Prevalence, intensity, and distribution of anisakid infection in *Decapterus* spp

**Figure 2:**
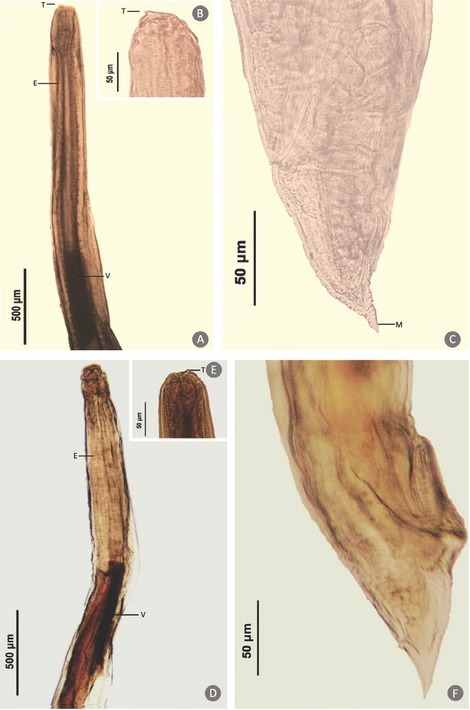
Light microscopic images of representative marine ascaridoid larvae collected from *Decapterus* spp. A and D, anterior part of the body, ventral view, showing the larval tooth (T), esophagus (E), and ventriculus (V); B and E (inset), detailed larval tooth (T); C and F, conical tail showing or lacking a mucron (M), respectively.

**Figure 3:**
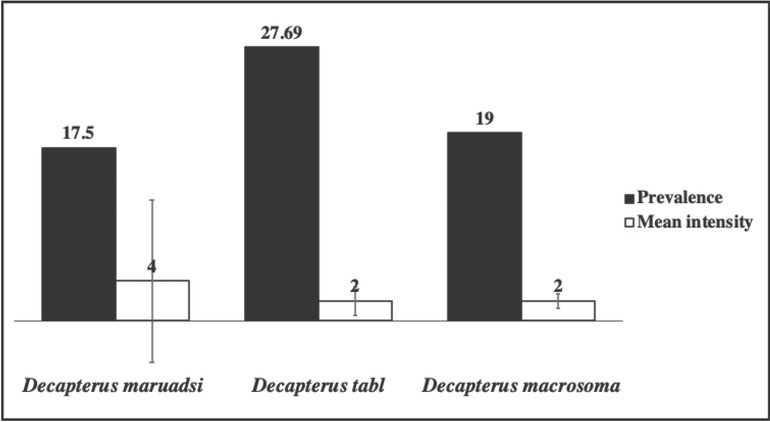
Prevalence (%) and mean intensity (worms/host ± SD) of marine ascaridoid larvae across the three *Decapterus* spp. (*N* = 371) collected from Tayabas Bay and Balayan Bay, southern Luzon, Philippines.

Three hundred seventy-one *Decapterus* spp. (mean weight: 68.59 ± 33.72 g; mean length: 18.56 ± 2.97 cm) morphologically identified as *D. maruadsi*, *D. tabl*, and *D. macrosoma* were collected from Tayabas Bay and Balayan Bay adjacent to the West Philippine Sea. Of the 371 fishes examined, 80 (22%) were infected with helminth parasites initially identified as marine ascaridoid larvae through visual examination. Macroscopically, the parasites were small, thin, whitish nematodes. Under the microscope, the body of the larvae was cylindrical in shape and attenuated at both ends. Shared characteristics among individual larvae are based on general morphology of the third-stage larvae of ascaridoid nematodes, which include a smooth cuticle with transverse striations, inconspicuous lips, presence of boring tooth at the anterior end, straight gut structure composed of a muscular esophagus, irregularly shaped ventriculus, an esophagus–intestinal junction which was poorly defined in some specimens, and a tail with or without a terminal mucron (Fig. 2). Among the fish species, *D. tabl* showed the highest marine ascaridoid larvae infection rate (27.69%), followed by *D. macrosoma* (19%), and *D. maruadsi* (17.50%) showed the lowest, as shown in Figure 3. Each of the 80 infected *Decapterus* spp. hosted from one to seven anisakid larvae, except for one specimen of *D. maruadsi* with 39 larvae recovered. Consequently, the highest intensity was observed in *D. maruadsi* (4 ± 8.24), followed by *D. tabl* (2 ± 1.42) and then by *D. macrosoma* (2 ± 0.73). Both prevalence (x^2^ [10] = 12.0, *P* = 0.285) and intensity (x^2^ [6] = 6.0, *P* = 0.423) of infection were not significantly different across fish host species.

**Figure 4:**
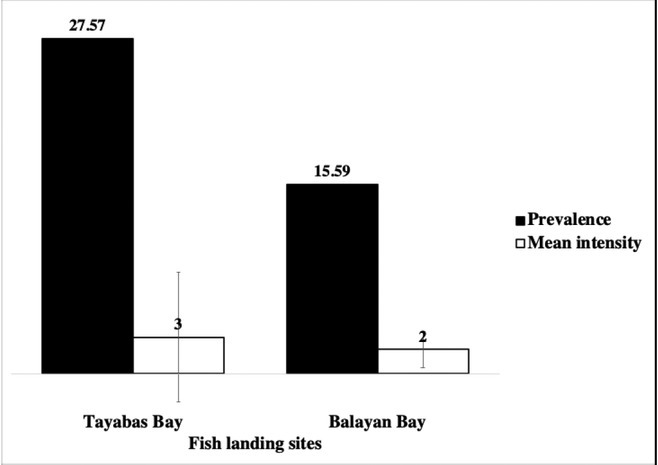
Prevalence (%) and mean intensity (worms/host ± SD) of marine ascaridoid larvae across the three *Decapterus* spp. (*N* = 371) collected from Tayabas Bay and Balayan Bay, southern Luzon, Philippines.

The variation in the prevalence and mean intensity of marine ascaridoid larvae with respect to the sampling areas is shown in Figure 4. A total of 185 individuals were collected from Tayabas Bay fish landing area in Quezon Province, while 186 individuals were from Balayan Bay in Batangas. Of the 371 fish samples, 51 (27.57%) were observed as infected from Tayabas Bay and 29 (15.59%) from Balayan Bay. While a higher prevalence is observed in Tayabas Bay than in Balayan Bay, there was a difference in the mean intensity of infection of fish samples collected from the two sites, 3 ± 5.33 in Tayabas Bay and 2 ± 1.49 in Balayan Bay. Statistical analysis revealed that prevalence (x^2^ [5] = 6.0, *P* = 0.306} and mean intensity (x^2^ [3] = 6.0, *P* = 0.112) did not vary significantly between the sites.

**Figure 5:**
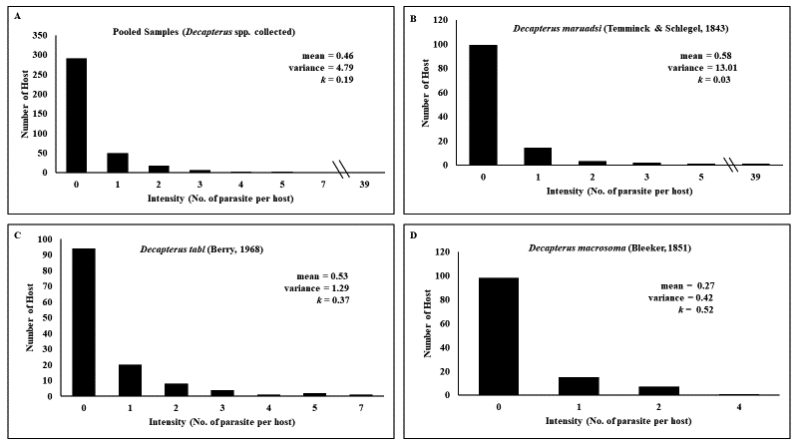
Aggregated distribution of marine ascaridoid larval parasites from (A) pooled *Decapterus* spp. samples, (B) *Decapterus maruadsi*, (C) *Decapterus tabl*, and (D) *Decapterus macrosoma* from Tayabas Bay and Balayan Bay, southern Luzon, Philippines.

The marine ascaridoid parasites showed an aggregated distribution in *Decapterus* spp., with the value of variance significantly greater than the mean (4.79 > 0.46). The resulting variance/mean ratio showed a significant departure from Poisson (random) distribution. A negative binomial distribution parameter was also used to estimate the degree of aggregation. The resulting measurement of aggregation (*k*) of the negative binomial revealed that the parasites are highly aggregated in distribution, where the value of *k* is <1 (*k* = 0.19) (Fig. 5).

### Molecular characterization and phylogenetic analysis

Evaluation of the identity of the marine ascaridoid larvae was performed using molecular analysis based on the 18S rRNA and the 5.8S-ITS2-28S markers. Twelve of the 15 selected larval samples generated DNA sequences that were subjected to subsequent molecular analyses. Single-gene alignment of both 18S rRNA and 5.8S-ITS2-28S regions identified two genera, *Anisakis* (*n* = 8) and *Raphidascaris* (*n* = 4). The closest species matches with the highest identity score for the ascaridoid larvae in the present study include *A. typica* (Deising, 1860) Baylis 1920, *Anisakis simplex* (Rudolphi, 1809), *Raphidascaris* (*Ichthyascaris*) *lophii* (Wu, 1949) Hartwich 1975, and *Raphidascaris* (*Ichthyascaris*) *longispicula* (Table 2). The partial sequences of 18S rRNA (628 bp) generated alignments, which presented genetic matches of the eight samples with zoonotic *Anisakis simplex* complex (99.20%–99.68%) and four others with *Raphidascaris longispicula* (99.52%–99.84%). Aligning the sequences using the 5.8S-ITS2-28S region (460 bp), the ascaridoid larvae were found to be genetically similar with *A. typica* (98.61%–99.88%) and *Raphidascaris lophii* (99.18%–99.79%).

**Table 2:** BLAST results for the 18S rRNA and 5.8S-ITS2-28S sequences of ascaridoid samples.

Specimen	18S rRNA		5.8S-ITS2-28S		Family
accession (18S/ITS2)	GenBank accession	% Match	GenBank accession	% Match	
OK659774/ OK659786	MF072704 (*Raphidascaris longispicula)*	99.84	MF422212 (*Raphidascaris longispicula)*	99.18	Raphidascarididae
OK659775/ OK659787	MF072711 (*Anisakis simplex*) MF072697 (*Anisakis pegreffii*) U94365 (*Anisakis* sp.)	99.52	HF911524 (*Anisakis simplex*)	99.88	Anisakidae
OK659776/ OK659788	MF072704 (*Raphidascaris longispicula*)	99.84	MF422212 (*Raphidascaris longispicula*)	99.79	Raphidascarididae
OK659777/ OK659789	MF072711 (*Anisakis simplex*) MF072697 (*Anisakis pegreffii*) U94365 (*Anisakis* sp.)	99.68	HF911524 (*Anisakis simplex*)	99.88	Anisakidae
OK659778/ OK659790	MF072704 (*Raphidascaris longispicula*)	99.52	MF422212 (*Raphidascaris longispicula*)	99.79	Raphidascarididae
OK659779/ OK659791	MF072704 (*Raphidascaris longispicula*)	99.52	MF422212 (*Raphidascaris longispicula*)	99.59	Raphidascarididae
OK659780/ OK659792	MF072711 (*Anisakis simplex*) MF072697 (*Anisakis pegreffii*) U94365 (*Anisakis* sp.)	99.20	HF911524 (*Anisakis simplex*)	98.66	Anisakidae
OK659781/ OK659793	MF072711 (*Anisakis simplex*) MF072697 (*Anisakis pegreffii*) U94365 (*Anisakis* sp.)	99.68	HF911524 (*Anisakis simplex*)	98.79	Anisakidae
OK659782/ OK659794	MF072711(*Anisakis simplex*) MF072697 (*Anisakis pegreffii*) U94365 (*Anisakis* sp.)	99.52	HF911524 (*Anisakis simplex*)	99.87	Anisakidae
OK659783/ OK659795	MF072711(*Anisakis simplex*) MF072697 (*Anisakis pegreffii*) U94365 (*Anisakis* sp.)	99.68	HF911524 (*Anisakis simplex*)	98.86	Anisakidae
OK659784/ OK659796	MF072711 (*Anisakis simplex*) MF072697 (*Anisakis pegreffii*) U94365 (*Anisakis* sp.)	99.68	HF911524 (*Anisakis simplex*)	98.61	Anisakidae
OK659785/ OK659797	MF072711 (*Anisakis simplex*) MF072697 (*Anisakis pegreffii*) U94365 (*Anisakis* sp.)	99.68	HF911524 (*Anisakis simplex*)	99.87	Anisakidae

ITS2, internal transcribed spacer 2.

**Figure 6:**
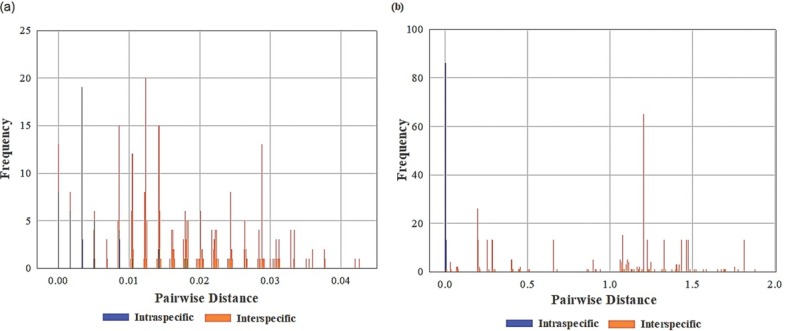
Intra- and interspecific sequence variations in Anisakidae and Raphidascarididae obtained in the GenBank and sample isolate sequences. (A) 18S rRNA and (B) 5.8S-ITS2-28S regions.

Pairwise distance comparison (Table 3) indicates low genetic variation within species for both 18S and 5.8S-ITS2-28S markers. However, sequence variation between species and between genera is at lowest for 18S rRNA gene, with *P*-distance ranging from 0 to 0.0425. Hence, an overlap for the pairwise comparison is observed between the paired entities for both intra- and interspecific categories (Fig. 6). A more pronounced overlap, however, is found among the 18S sequences of the GenBank and sample isolates. Meanwhile, a higher variability in the sequences of the different species and genera is observed for the 5.8S-ITS2-28S region (*P*-distance 0.004 to 1.877).

**Figure 7:**
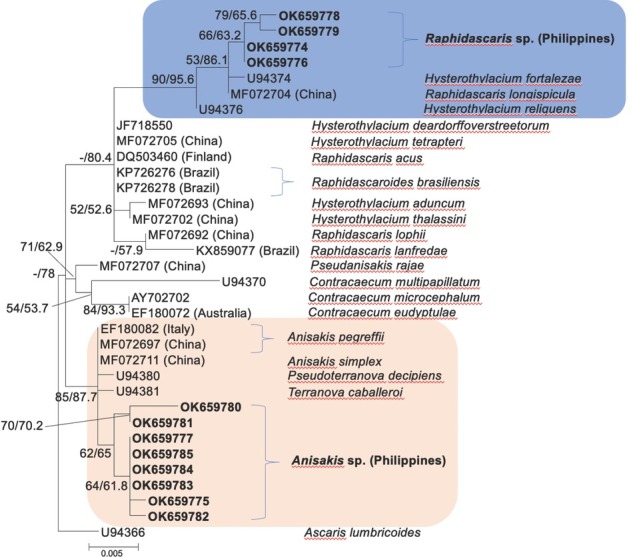
ML phylogenetic tree of Anisakidae and Raphidascarididae inferred from 628 nucleotide positions of the 18S rRNA gene using the TrNef + G model of DNA substitution and rooted on *Ascaris lumbricoides* (U94366). Values on nodes represent ML/NJ bootstrap percentages of 1,000 bootstrap samples; values <50% are not shown. Scale bar represents five nucleotide substitutions of 1,000 nucleotides. Sequences from this study are highlighted. ML, maximum-likelihood; NJ, neighbor-joining.

Phylogenetic reconstruction using the ML and NJ methods based on the 18S rRNA gene showed two major clusters of the currently studied ascaridoid larvae. Four samples were resolved in close association with *Raphidascaris longispicula* and *Hysterothylacium* species, with a bootstrap percentage of 90/95.6% ML/NJ bootstraps (Fig. 7). Meanwhile, the other eight samples were grouped together, forming an external cluster with the zoonotic *A. simplex* species complex (85/87.7% ML/NJ bootstraps). In this reconstruction, *A. typica* species was not represented due to the unavailability of GenBank accessions.

**Table 3:** Corrected (TrNef + G) genetic distances of 18S rRNA gene and corrected (HKY + G) genetic distances of the 5.8S-ITS2-28S region of Anisakidae and Raphidascarididae nematodes.

Ascaridoid comparisons	Number of pairwise comparisons	18S rRNA		5.8S-ITS2-28S region	
		Minimum	Maximum	Minimum	Maximum
**Intraspecific**	67 (18S), 118 (ITS2)	0	0.0182098	0	0.0077422
**Interspecific**	494 (18S), 410 (ITS2)	0	0.04259699	0.00493606	1.8776778

ITS2, internal transcribed spacer 2.

**Figure 8:**
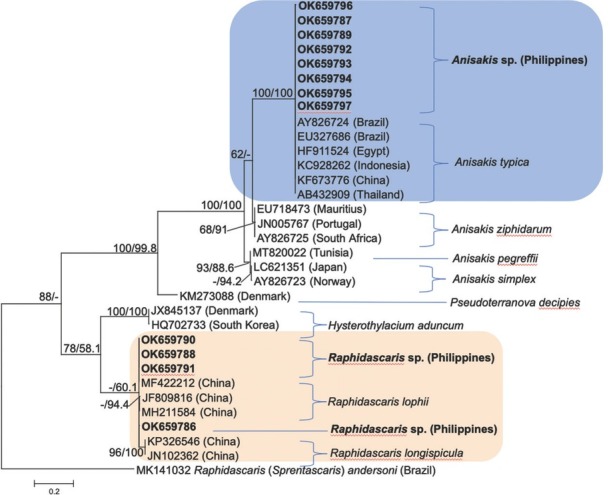
ML phylogenetic tree of Anisakidae and Raphidascarididae inferred from 460 nucleotide positions from the partial sequence of the 5.8S, whole sequence of the ITS2, and partial sequence of 28S rRNA gene using the HKY + G model of DNA substitution and rooted on *Raphidascaris* (*Sprentascaris*) *andersoni*. Values on nodes represent ML/NJ bootstrap percentages of 1,000 bootstrap samples; values <50% are not shown. Scale bar represents two nucleotide substitutions of 10 nucleotides. Sequences from this study are highlighted. ITS2, internal transcribed spacer 2; ML, maximum-likelihood.

The phylogeny based on the partial sequence of the 5.8S-ITS2-28S rRNA region (Fig. 8) showed similar grouping of the four *Raphidascaris* sp. larval samples as an external sister clade of *R. lophii* with strong nodal support for both ML and NJ trees (96/100% bootstraps). The inclusion of *A. typica* reference accessions generated similar clustering of *Anisakis* sp. samples with full support (100/100% ML/NJ bootstraps). It can be inferred that all *Anisakis* sp. samples are genetically similar to *A. typica* (*P*-distance = 0.0077422) of cosmopolitan distribution.

## Discussion

This study surveyed *Decapterus* spp. (scad fish), which include the shortfin scad (*Decapterus macrosoma* Bleeker, 1851), Japanese scad (*D. maruadsi* Temminck and Schlegel, 1843), and roughear scad (*D. tabl* Berry, 1968). *Decapterus macrosoma* and *D. maruadsi* comprise a major portion of the total *Decapterus* fish catch in the Philippines and are economically important with high commercial value. However, the Bureau of Fisheries and Aquatic Resources of the Department of Agriculture (BFAR-DA) in the Philippines recognized roughear scad as one of the species subjected to heavy exploitation by commercial fishing. Collectively, *Decapterus* species is ranked first in the production of commercial fisheries in the country (Narido et al., 2016). The decline of fish populations can be attributed to several stressors, including overfishing, highly efficient technologies, bycatch, and overcapacity (Cudmore, 2009). Other factors such as temperature, environmental pollution, and parasitic infection can also affect the fish population.

Early reports on anisakid infection in marine fish showed that the parasites infect muscles around the belly area of fish (Petersen et al., 1993). This study also harvested the parasites from the abdominal cavity, stomach, and intestine of host fish. This is similar to the findings of various studies showing the gastrointestinal tract to be the preferred site among parasitic helminths (Gosselle et al., 2008; Onyedineke et al., 2010; Dauda et al., 2016). It is important to note that the method implemented for isolation and examination of the parasites was the common routine visual observation coupled with identification based on taxonomic descriptions from previous studies. However, the major disadvantage of this method is the tendency of missing out parasites with predilection sites other than the gastrointestinal tract, especially those that are embedded within tissues, which were not covered in this study. Another simple method was explored by various researchers, such as Shamsi and Suthar (2016), which will be beneficial if considered. Here, it was initially assumed that the helminth parasite larvae were identified as marine ascaridoid parasites based on the morphological characteristics. The results revealed that 22% of the fishes were detected with the parasite, with infection rates not significant between male and female fish.

Aggregation analysis revealed a highly aggregated distribution of parasites in the host population, with *D* = 0.86, *k =* 0.19 (Fig. 5). It demonstrated that only a few members of *Decapterus* spp. in the population exhibited high infection rates and that a majority had low to moderate infections. This result proved that the parasites infect several fish hosts and maintain the host population by not overburdening the parasite load in most host individuals. Only very few hosts harbor a great number of parasites, thus ensuring that the host population is intact and healthy and will still be available for future parasite life cycles. Parasite aggregation is important to study in host–parasite ecology since it has a direct effect on host fitness and the stability of the host population dynamics (Estaño et al., 2021). Thus, the commonly observed overly dispersed distribution of parasite populations may contribute to their survival, reproductive success, and survival of the fish host population (Anderson and Gordon, 1982; Calabrese et al., 2011). This tends to stabilize host–parasite dynamics, showing only a few infected among the susceptible hosts available for infection.

Only a limited number of larvae were subjected to molecular analysis. We speculated that these larvae belong to the same taxonomic group due to gross morphological similarities. However, morphological characterization of ascaridoid larvae poses difficulty in identifying species, particularly from families with cryptic larval features. Although morphological characteristics are useful for species description and identification, the characterization may be limited because many macroscopic structures are produced infrequently and microscopic features of ascaridoid larvae are similar across different species (Mattiucci and Nascetti, 2006; Quiazon et al., 2008; Cavallero et al., 2014). Identifying which parasite species infects the fish populations is imperative since there are occurrence of zoonotic parasites that may affect the health of fish-eating populations.

Different genera of Anisakidae have been described using molecular tools more accurately than using classification based on their morphological characteristics and can be classified into either family Anisakidae or family Raphidascarididae (Kalay et al., 2009). Phylogenetic analyses based on the 5.8S-ITS2-28S DNA region confirmed close association of *Anisakis* samples with *A. typica* reference sequences and the *Raphidascaris* samples with *Raphidascaris lophii*. In this study, phylogenetic trees constructed based on the 5.8S-ITS2-28S marker resolved the clustering of the ascaridoid larvae with the GenBank sequences better than the 18S rRNA sequences. The ITS2 segment of the nuclear rDNA has long been used as an ideal DNA barcode because of its short length, easy amplification, and high sequencing efficiency. Unlike the highly conserved 18S gene, the ITS2 marker exhibits high variability to distinguish closely related species (Yao et al., 2010; Yu et al., 2017). This interspecific variation makes ITS a suitable marker for identifying cryptic species such as those in *Anisakis* (Cavallero et al., 2014). Furthermore, Malta et al. (2018) mentioned that phylogenetic resolution becomes better with more taxa included in the analysis. Since there are more species matches obtained using 5.8S-ITS2-28S than the 18S gene, it becomes more informative, showing all morphologically similar samples together in one cluster with strong nodal support. This is not observed in the phylogenetic reconstruction using 18S. Abouheif et al. (1998) have regarded 18S as unsuitable for reconstructing the evolutionary history of metazoan phyla. The poor resolution of phylogeny using 18S may be attributed to it being highly conserved that it cannot resolve robust phylogenies. Also, there is a low number of reference sequence submissions that represent the taxa in question (Safonova et al., 2021).

*Anisakis* are known to have a wide geographic distribution across all continents (Pampiglione et al., 2002). These species are mostly reported as parasites of various dolphin species in warmer temperate and tropical waters. Mattiucci et al. (2002) have cited the first attempt of Bagrov (1982) in linking the larvae from *Gempylus serpens* (Cuvier, 1829) in Philippine waters with *A. typica* but presented no evidence for the identification. Recent records of *Anisakis* species in the Philippines were those retrieved from the whales from southern Philippines (Quiazon et al., 2013, Quiazon et al., 2021). To date, there are very few studies on the marine fish parasites in the Philippines. The recent survey conducted by the BFAR in Region 4A on different marine fishes presented anisakid and other endoparasitic nematodes infecting commercially important fish species such as *Rastrelliger kanagurta* (Cuvier, 1816), *Sardinella lemuru* (Bleeker, 1853), *Atule mate* (Cuvier, 1833), and *Selar crumenophthalmus* (Bloch, 1793). These fishes were also caught in Tayabas Bay. Moreover, these *Anisakis* species have a documented distribution in the South China Sea, East China Sea, Atlantic Ocean, and eastern Mediterranean Sea (WoRMS Editorial Board, 2021). It is then most likely that parasites such as *Anisakis* and *Raphidascaris* are present in Tayabas Bay and Balayan Bay, which are in the southeastern region of the West Philippine Sea.

The study revealed the presence of *A. typica* and *Raphidascaris lophii* in populations of *Decapterus* caught in Philippine waters based on morphological examination and phylogenetic analyses. This is a first report on the molecular identification of *Anisakis* and *Raphidascaris* parasites infecting some economically important fish species. To note, *Anisakis* parasites cause human anisakiasis. While the species, *A. typica*, recovered from this study has not been recorded as a public health concern in terms of causing human anisakiasis, their presence in fish that are a major part of the Filipino diet may cause potential health hazards. Moreover, albeit the low risk of human infection by *A. typica*, their predominance in fishes commonly consumed by people may present a potential outbreak of human cases for foodborne allergies at the very least (Umehara et al., 2010). Artificial infection of *A. typica* caused severe tissue damage in the stomach of rats as such, and this species can potentially impact animal and human health alike (Soewarlan et al., 2015; Tunya et al., 2020). Certain caution must be taken, especially in areas where raw fish consumption is customary like in the Philippines. Moreover, paucity in the database of Philippine marine fish parasites warrants more research efforts, especially concerning economically important fish species with implications to food safety and food security.
